# Isolation and screening of multifunctional phosphate solubilizing bacteria and its growth-promoting effect on Chinese fir seedlings

**DOI:** 10.1038/s41598-021-88635-4

**Published:** 2021-04-27

**Authors:** Jiaqi Chen, Guangyu Zhao, Yihui Wei, Yuhong Dong, Lingyu Hou, Ruzhen Jiao

**Affiliations:** 1 Research Institute of Forestry, Chinese Academy of Forestry, Beijing, 100091 China; 2 State Key Laboratory of Tree Genetics and Breeding, Chinese Academy of Forestry, Beijing, 100091 China; 3 Key Laboratory of Tree Breeding and Cultivation of State Forestry Administration, Chinese Academy of Forestry, Beijing, 100091 China

**Keywords:** Ecology, Microbiology, Ecology

## Abstract

Phosphorus-solubilizing microorganisms is a microbial fertilizer with broad application potential. In this study, 7 endophytic phosphate solubilizing bacteria were screened out from Chinese fir, and were characterized for plant growth-promoting traits. Based on morphological and 16S rRNA sequence analysis, the endophytes were distributed into 5 genera of which belong to *Pseudomonas*, *Burkholderia*, *Paraburkholderia*, *Novosphingobium*, and *Ochrobactrum.* HRP2, SSP2 and JRP22 were selected based on their plant growth-promoting traits for evaluation of Chinese fir growth enhancement. The growth parameters of Chinese fir seedlings after inoculation were significantly greater than those of the uninoculated control group. The results showed that PSBs HRP2, SSP2 and JRP22 increased plant height (up to 1.26 times), stem diameter (up to 40.69%) and the biomass of roots, stems and leaves (up to 21.28%, 29.09% and 20.78%) compared to the control. Total N (TN), total P (TP), total K (TK), Mg and Fe contents in leaf were positively affected by PSBs while showed a significant relationship with strain and dilution ratio. The content of TN, TP, TK, available phosphorus (AP) and available potassium (AK) in the soil increased by 0.23–1.12 mg g^−1^, 0.14–0.26 mg g^−1^, 0.33–1.92 mg g^−1^, 5.31–20.56 mg kg^−1^, 15.37–54.68 mg kg^−1^, respectively. Treatment with both HRP2, SSP2 and JRP22 increased leaf and root biomass as well as their N, P, K uptake by affecting soil urease and acid phosphatase activities, and the content of available nutrients in soil. In conclusion, PSB could be used as biological agents instead of chemical fertilizers for agroforestry production to reduce environmental pollution and increase the yield of Chinese fir.

## Introduction

Phosphorus is one of the important nutrients that affect plant growth and metabolism^1^, and it is also a limited natural resource, which is gradually being recognized as a new challenge for global sustainable development^2, 3^. Because available phosphorus is not enough to meet the needs of plant growth^4^. Especially the subtropical forest soil is severely deficient in phosphorus^5, 6^, which greatly limits the development and productivity of plants. About 52.3 billion tons of phosphorus fertilizer are used annually to maintain soil fertility. However, The recovery of P fertilizer in the soil is quite low (15–20%), and the residual P is fixed in soils^7^. Therefore, improving the absorption and use of P by crops is of great significance from both the ecological and economical perspective^8^.

Phosphate solubilizing bacteria (PSB) are a group of microorganisms that are capable of solubilizing insoluble phosphate, fixing nitrogen and secreting auxin, thereby promoting plant growth. In recent years, working on PSB mainly focused on plant growth^9, 10^. It has shown that seed or soil inoculated with phosphate-solubilizing bacteria (PSB) is known to improve the solubilization of fixed soil phosphorus and applied phosphates, resulting in higher performance of plants^11, 12^. For instance, Hansen et al.^13^ inoculated Bacillus simplex into winter wheat plants, which significantly increased the nutrients of plant, such as P concentration in root biomass and concentrations of Mg, Mn and S in shoot biomass. Tahir et al.^14^ found that inoculation with PSB significantly increased wheat growth rate, phosphorus uptake, and wheat yield increased by 10–12%. Ahmad et al.^15^ isolated the PSB PSB1, PSB2, and PSB7 from the rhizosphere of *Zea mays* and these were shown to promote dry matter yield as compared to the control. High-efficiency PSB have the potential for making a great contribution to the decrease of environmental pollution and promoting ecological balance by replacing chemical fertilizers^16^.

Due to the influence of indigenous microorganisms, soil environment and other factors, the phosphorus-solubilizing microorganisms in the soil are difficult to colonize successfully, resulting in unstable application effects^17, 18^. As an important part of the entire micro-ecosystem of plant plants, endophytes have multiple functions such as nitrogen fixation, enhancement of plant resistance, resistance to pests and diseases, and promotion of plant growth^18^; at the same time, most plant endophytes are facultative endophytes, and they not only exist in plants, but are also common in rhizosphere soil and other environments^19^. Chinese fir (*Cunninghamia lanceolata*) is the timber tree species with the largest afforestation area in southern China^20^. In recent years, due to the intensive management of fir and the shortened rotation period, the productivity of fir forests has declined^21^. In addition, the high iron and aluminum content of the soil in southern areas and strong leaching can easily cause the fixation and inactivation of phosphorus in the soil^22^, resulting in a very low utilization rate of soil phosphorus, which seriously limits the sustainable development of Chinese fir plantation. Therefore, it is of great significance to study the effect of phosphorus-solubilizing bacteria on the growth of Chinese fir for the future application of PSB on forest trees.

In this study, phosphorus-solubilizing bacteria were isolated and screened from the roots, stems, and leaves of Chinese fir, through research on the growth-promoting properties of nitrogenase activity, 1-Aminocyclopropane-1-Carboxylate (ACC) deaminase activity, Indole-3-Acetic Acid (IAA) production and siderophore. Here, we aimed to taking Chinese fir seedling as the material and investigating the effects of inoculation with PSB on the plant growth, nutrient content soil enzyme activity. The results of this study will provide basic data and practical guidance for the development and applications of PSB as biofertilizers in forestry.

## Materials and methods

### Plant materials

In April 2019, 2-year-old Chinese fir seedlings were collected from the Yalin Center of the Chinese Academy of Forestry in good condition and free from pests and diseases. (The use of Chinese fir seedlings in the experiment complies with national regulations).

### Medium

Pikovskava (PVK) solid medium: glucose 10 g, Ca_3_(PO_4_) 25 g, CaCO_3_5g, (NH_4_)_2_SO_4_0.5 g, NaCl 0.2 g, MgSO_4_ 7H_2_O 0.1 g, KCl 0.1 g, MnSO_4_ 0.002 g, FeSO_4_ 7H_2_O 0.002 g, agar 18 g, Distilled water 1000 mL, pH 7.0.

PVK liquid medium: PVK solid medium without agar.

Luria-Bertan (LB) medium: tryptone 10 g, yeast extract powder 5 g, NaCl 10 g, agar 18 g, distilled water 1000 mL, pH7.0.

LB liquid medium:LB solid medium without agar.

### Isolation and purification of endophytes from Chinese fir seedlings

The roots, stems, and leaves of the selected Chinese fir seedlings were washed away with running water to remove the surface soil, and then washed with running water for 24 h to 36 h, and the surface moisture was absorbed with sterile filter paper. Weigh 1 g of roots, stems and leaves in a petri dish, and then carry out surface disinfection in a sterile operating table with 75% alcohol (C_2_H_5_OH) for 30 s, 5% sodium hypochlorite (NaClO) 10 min, wash the sterile water 7 times, and use sterile filter paper to absorb the water. The material after surface sterilization is cut into 2 mm × 2 mm with sterile surgical scissors, placed in a sterilized mortar and grated with a small amount of sterile quartz sand and ground into a homogenate, then diluted with sterile water to 10^–1^, 10^–2^ and 10^–3^, pipette to draw 150 µL of sample grinding fluid, spread on the medium, set the sterile water of the last rinse of Chinese fir tissue as a blank control, incubate with other plates under the same conditions, and verify whether the surface disinfection. Cultivate in a 28 ℃ incubator according to the characteristics of the colony phenotype, and use the streak separation method to further purify and isolate the strains until the isolation of the colony morphology is uniform for each isolate.

Note: 75% C_2_H_5_OH: 75 mL absolute ethanol + 25 mL sterile deionized water; 5% NaClO: sodium hypochlorite solution with 10% available chlorine: sterile deionized water = 1:1.

### Characterization of PGP traits

#### Determination of phosphorus solubilizing ability

The strain was inoculated into PVK liquid medium and cultured at 28 ℃ for 180 days/min on a reciprocating shaker for 7 days, and then the pH value of the medium was measured. The culture solution was centrifuged at 8000 rpm for 15 min to remove bacterial cells. Take the supernatant and use the molybdenum antimony scandium colorimetric method^23^ to determine the soluble phosphorus content in the culture broth.

#### Determination of nitrogenase activity

An aliquot of 200 µL fresh culture was inoculated to 20 mL of nutrient broth and incubated overnight at 30℃. Bacterial growth was collected by centrifugation and was washed twice using sterile water, and resuspended by liquid limited nitrogen culture medium (OD_600_ = 0.2). The 3 mL suspension was transferred to a 25 mL sterilized serum vial and 2.4 mL acetylene gas (99.9999%) was driven into the serum bottle, and then incubated at 30 °C for 12 h. The ethylene content and the protein of bacterial suspension were determined as You et al.^24^.

#### 1-Aminocyclopropane-1-carboxylate (ACC) deaminase activity determination

ACC deaminase activity was determined by the method of Glick et al.^25^ using N-free medium (Nfb)^26^ for bacteria and minimal medium (MM)^27^ for actinomycetes containing 0.3 m mol L^−1^ ACC (Sigma, USA) as a sole nitrogen source. MM with 0.1% (w/v) NH_4_(SO_4_)_2_ was used as a positive control and cultivation without ACC was used as a negative control. After incubation at 28 ℃ for 7 days for non-actinomycete bacteria and 14 days for actinomycetes, colony growth on Nfb or MM with addition of ACC indicated ACC deaminase activity.

#### Indole-3-acetic acid (IAA) production

IAA production was measured by colorimetric assay^27^. Bacterial isolates were cultured for 3 days in TY broth (without L-tryptophan or supplemented with 500 μg/mL of l-tryptophan) in the dark at 28 °C. Cells were removed from the culture medium by centrifugation at 13,000×*g* for 10 min; then, 1 mL of the supernatant was mixed vigorously with 2 mL of Salkowski’s reagent (4.5 g of FeCl_3_ per L in 10.8 M H_2_SO_4_). Samples were incubated at room temperature for 30 min and the IAA production was estimated from the optical density at 600 nm (OD_600_) by comparison with a standard curve prepared from known concentrations of IAA.

#### Siderophore production

Siderophore production was examined by using chrome azurol S (CAS) agar^28^. Isolate was inoculated onto CAS agar, cultured at 28 °C for 2 days, and the positive strain was indicated by an orange halo around the bacterial colony. Determine the ratio (D/d) of orange aperture diameter (D) to colony diameter (d) to determine the iron-producing carrier capacity of the strain.

#### Physiological and biochemical tests of phosphate-solubilizing bacteria

The conventional physiological and biochemical identification of PSB is carried out according to the methods in the “Common Bacterial System Identification Manual”, which mainly includes Gram stain, glucose hydrolysis test, lactose hydrolysis test, methyl red test, Voges-Proskauer (VP) test, hydrogen sulfide production test, gelatin liquefaction Test, citrate utilization test, malonate utilization test, denitrification test.

#### 16 SrRNA gene sequencing

Taking the screened multifunctional PSB as the object, the bacterial genomic DNA extraction kit of Beijing Bomed Biotechnology Co., Ltd. was used to extract the DNA of the strain, using the DNA as a template, and using the bacterial universal primer 27F (5′-AGAGTTTGATCCTGGCTCAG-3′) and 1492R (5′-GGTTACCTTGTTACGACTT-3′) PCR amplification, the amplification system is as follows: DNA template 1 uL, primer 27F 0.5 µL, 1492R 0.5 µL, 2 × TaqMix 12.5 µL, ddH_2_O10.5 µL. The PCR procedure is as follows: 93℃ for 3 min, 93℃ for 30 s, 56 ℃ for 30 s, 72℃ for 2 min, 32 cycles; 72 ℃ for 7 min. The amplified products were sequenced bidirectionally by BGI. After splicing the measured 16SrDNA sequences in ContigExpress, search in GenBank, EzTaxon, BIGSdb databases respectively, select the model strains with high homology. The phylogenetic tree was constructed by the neighbor-joining method using MEGA version 7.0 with the Kimura 2-parameter model^29^, the robustness of the tree was evaluated by performing bootstrap analyses based on 1000 replications^30^.

### Evaluation of plant growth promotion by individual inoculation

#### Preparation of inoculum

The single colonies were picked out and incubated in LB broth at 180 rpm at 28 ℃ for 12 h. The above solution was inserted into 200 mL of LB broth at 1% inoculation, incubated at 180 rpm at 28℃ for 48 h and the cell pellet was resuspended in sterile distilled water and made up to a final concentration of 3 × 10^8^ CFU/mL.

#### Experimental seedlings and soil

Chinese fir seedlings were provided by the Experimental Center of Subtropical Forestry, Chinese Academy of Forestry (117° 67′ E, 27° 82′ N), Jiangxi Province, China. The use of Chinese fir seedlings in the experiment complies with national regulations. Five months old seedlings with vigorous and apparently disease and pest free were used. The height and root collar diameters of seedlings were 8.7 cm and 1.36 mm, respectively. The seedling container was made of non-woven fabric, the specification was 4.5 cm × 8.0 cm (Diameter × Height). The soil was consisted of nursery medium and loess at a ratio of 9:1, was thoroughly mixed and homogenized with 3 kg slow-release fertilizer per cubic. The slow-release fertilizer is produced by American Abbes (180 g kg^−1^ total N, 80 g kg^−1^ available P, and 80 g kg^−1^ total K, the fertilizer effect period is 9 months). The soil exhibited the following properties: 6.34 g kg^−1^ total N, 0.80 g kg^−1^ total P, 2.50 g kg^−1^ total K, and a pH-value of 6.00.

#### Test design

The SSP2, JRP22 and HRP2, which were confirmed to have the characteristics of promoting plant growth, so pot experiments were conducted. To determine the effectiveness of phosphorus-solubilizing bacteria in plant growth of Chinese fir, a pot culture experiment was conducted between August and November 2019 in an open-sided greenhouse in Experimental Center of Subtropical Forestry, Chinese Academy of Forestry, Jiangxi Province, China. The experiment was carried out in three-factor orthogonal design with five replications for each treatment. The orthogonal experimental design of experiment is provided in Table 1, and strain, dilution ratio, inoculation method contained 3 levels. The pots with water was used as control (CK). For the irrigation of the rhizosphere (IR) treatments, 30 mL of diluted inoculum was added to the soil in the vicinity of the roots of Chinese fir. For the foliar spray (FS) treatments, 30 mL diluted bacterial cell suspension was inoculated in the leaves of Chinese fir by using a syringe. For the rhizosphere + foliar spray (IS) treatments, 15 mL of diluted inoculum was inoculated in the rhizosphere of seedlings, 15 mL was added to the leaves of seedlings by foliar spray. Each treatment contained sixteen seedlings for a total of nine treatments. A total of 3 inoculations were given in the middle of each month. The plant height and stem diameter were recorded before the first inoculation. The plants were harvested after 90 days (16 plantlets/replicate/treatment, i.e., a total of 80 plantlets per treatment) and the root biomass, stem biomass, leaf biomass, plant height and stem diameter were measured.

**Table 1:** L_9_(3^4^) Orthogonal design of experiment.

Treatment	Strain	Dilution ratio	Inoculation method
T1	SSP2	1:30	IR
T2	SSP2	1:60	FS
T3	SSP2	1:90	IS
T4	JRP22	1:30	FS
T5	JRP22	1:60	IS
T6	JRP22	1:90	IR
T7	HRP2	1:30	IS
T8	HRP2	1:60	IR
T9	HRP2	1:90	FS
CK	–	–	IR

*IR* irrigation of the rhizosphere, *FS* foliar spray, *IS* irrigation of the rhizosphere + foliar spray.

#### Determination of growth indicators

During the test, before each inoculation, the height of the seedlings was measured with a ruler and the ground diameter of the seedlings was measured with a vernier caliper. After the experiment, 10 plants of Chinese fir seedlings in average growth were randomly selected from each treatment, a total of 30 plants were washed with clean water to remove surface impurities, the filter paper was dried and the roots, stems, and leaves were put into paper bags respectively at 105 °C. After being degraded for 0.5 h, dried at 70 °C to a constant weight, weighed and recorded the biomass of each part.

#### Determination of leaf and soil nutrient content

Total N content of leaf and soil was measured by a 2300 Kjeltec Analyzer Unit (FOSS, Höganäs, Sweden). Total P and total K, total Mg and total Fe of leaf, soil TP, TK, AP and AK were extracted according to literature^31^ and were determined by ICP (Kleve, Germany).

#### Determination of soil enzyme activities

Activities of the soil urease, cellulase, sucrase, dehydrogenase and acid phosphatase were determined by spectrophotometry. Firstly, 0.05 g of soil was added to 450 mL of phosphate buffer solution (PBS, 0.1 mol L^−1^, pH 7.4). Then the solution was mixed by shaking, and centrifuged at 2000 rpm at 4 ℃ for 10 min and supernatant was collected with a new centrifugal tube. The supernatant and reagents were added according to the kit instructions (Shanghai Enzyme-linked Biotechnology Co., Ltd., Shanghai, China). Absorbance at 450 nm was measured on a SpectraMax Paradigm Multi-Mode detection platform (Molecular devices, San Jose, CA, USA).

#### Data processing and analysis

All statistical analyses were performed using SPSS24. Data are presented in terms of means (± SE; standard error). Statistical differences were tested by one-factor ANOVA to evaluate the differences in the nutrient content of soil and plant growth status. In MEGA7.0, the Neighbor-Joining method was used to construct the phylogenetic tree, and the Bootstrap value was 1000.

## Results

### Colony characteristics of phosphate-dissolving bacteria

Seven strains of putative phosphate-solubilizing bacteria were isolated and purified from Chinese fir seedlings, five were from the root system of Chinese fir plants, and one was isolated from the stems of Chinese fir plants and one was from leaves. Morphological characterization of bacterial colonies generally includes the observation of shape, color, surface texture, opacity, etc. These morphological characteristics of the seven isolated strains of putative phosphorolytic bacteria were observed and recorded (Table 2). It can be seen from Table 2 that most of the bacterial colonies were white or light yellow, round, and with a wet surface, while a few were transparent.

**Table 2:** Colony characteristics of phosphate solubilizing bacteria grown in PVK medium.

Strain no	Isolated part	Shape	Color	Surface state
HRP2	Stem	Round	Light yellow	Moist
JLP2	Leaf	Round	White	Dry
SSP2	Root	Round	White	Dry
JRP13	Root	Round	White	Moist
JRP22	Root	Round	Light yellow	Moist
JRP23	Root	Round	Light yellow	Dry
JRP24	Root	Round	White	Moist

### Physiological and biochemical tests

The physiological and biochemical characteristics of the selected PSBs are shown in Table 3. The screened strains were Gram negative and could all hydrolyze glucose. On the contrary, only JRP23 could hydrolyze lactose and produce hydrogen sulfide; however, it was also the only strain to score negative in the citrate test. Only JLP2 was methyl-red positive, and SSP2, JRP22, and JRP23 were methyl-red negative. Only JLP2, SSP2, and JRP22 showed a positive Voges–Proskauer (VP) test result. Only JRP23 and JRP24 failed to hydrolyze gelatin. Only JRP22 scored positive in the malonate test. HRP2, JLP2, JRP23, and JRP24 could not perform denitrification. Among these strains, JLP2, SSP2, and JRP22 exhibited similar physiological and biochemical characteristics; they could all use glucose and lactose as energy sources and scored negative in the methyl-red test and positive in the VP test. However, we could not classify strains according to their physiological and biochemical features; therefore, we carried out molecular identification to determine their respective genus and species.

**Table 3:** Physiological and biochemical features of phosphate-solubilizing bacteria.

Test	HRP2	JLP2	SSP2	JRP13	JRP22	JRP23	JRP24
Gram staining	−	−	−	−	−	−	−
Glucose hydrolysis	+	+	+	+	+	+	+
Lactose hydrolysis	+	+	+	+	+	−	−
Methyl red	+	−	−	+	−	−	+
V-P test	−	+	+	-	+	−	−
Hydrogen sulfide production	−	−	−	−	−	+	−
Gelatin liqueaction	+	+	+	+	+	−	−
Citrate	+	+	+	+	+	−	−
Malanate	−	−	−	−	+	−	−
Denitrification	−	−	+	+	+	−	−

### 16S rRNA gene sequences

**Figure 1:**
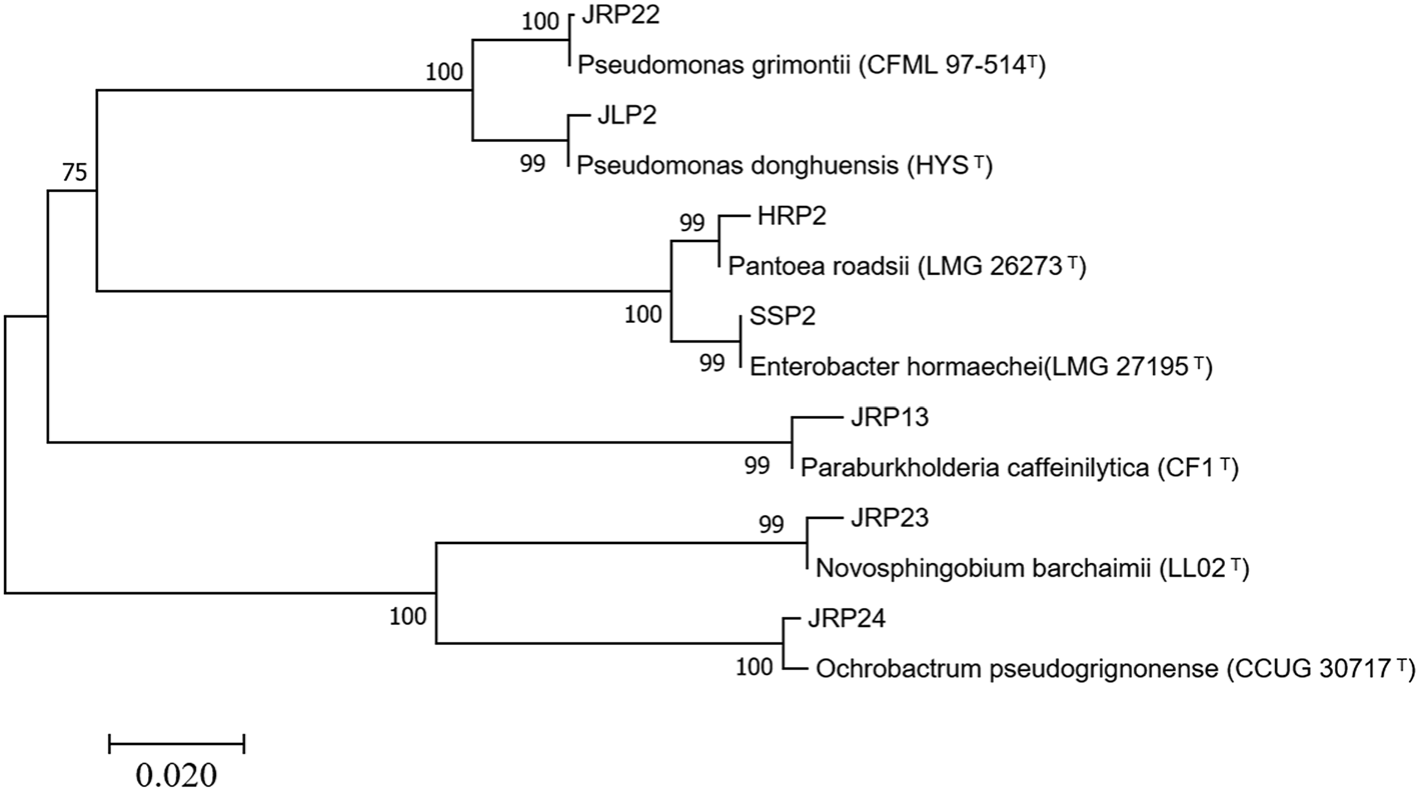
Phylogenetic evolutionary tree based on 16S rRNA sequences.

The nucleotide sequences of the 16S rRNA genes of the seven selected strains, approximately 1350 bp long, were amplified by PCR and sequenced. These sequences were compared by BLAST with those in the GenBank, EzTaxon, and BIGSdb databases, and the results are shown in Table 4. These results showed that the seven strains of growth-promoting bacteria belonged to six genera. In particular, two strains (JLP2 and JRP22) displayed high sequence similarity with *Pseudomonas* (98.75–100%). Moreover, the similarity between the 16S rRNA gene sequence of strain HRP2 and that of the genus *Pantoea* was 98.84%. The remaining four strains (SSP2, JRP13, JRP23, and JRP24) showed high sequence similarity with the genera *Enterobacter, Paraburkholderia*, *Novosphingobium*, and *Ochrobactrum*, respectively. In addition, the 16S rRNA gene sequence of strain JRP22 was identical to that of *Pseudomonas grimontii* (CFML97-514). Therefore, strain HRP2, displaying the strongest phosphate solubilization ability among these bacteria, belongs to the genus *Pantoea*, while the next most efficient phosphate-solubilizing bacteria, SSP2 and JRP22, belong to the genus *Enterobacter* and *Pseudomonas*. Using MEGA7.0 software, Neighbor-Joining was used to construct a phylogenetic tree of phosphate solubilizing bacteria (Fig. 1) to determine the type of each strain. The phylogenetic tree in the figure only shows values with a self-extension value greater than 70.

**Table 4:** Identification of PSB based on their 16S rRNA gene sequences.

Strain no.	Type strain	Identity (%)	Accession number
JLP2	*Pseudomonas donghuensis*(HYS)	99.73	NR136501.2
HRP2	Pantoea roadsii(LMG26273)	98.84	NR114154.1
SSP2	*Enterobacter hormaechei*(LMG 27195)	99.86	NR126208.1
JRP13	*Paraburkholderia caffeinilytica*(CF1)	99.31	NR152088.1
JRP22	*Pseudomonas grimontii*(CFML97-514)	100.00	NR025102.1
JRP23	*Novosphingobium barchaimii*(LL02)	99.69	NR118314.1
JRP24	*Ochrobactrum pseudogrignonense*(CCUG30717)	100.00	NR042599.1

### Growth-promoting traits of phosphate-solubilizing bacteria

To identify possible growth-promoting traits of the isolated bacteria, we screened them for several PGP-associated enzymatic activities or for the production of PGP compounds (Table 5). The results showed that three strains of PSB displayed nitrogenase activity, accounting for 40% of the tested strains, which led to C_2_H_2_ reduction rates ranging from 73.19 to 83.06 nmol mL^−1^ h^−1^. Among these, the HRP2 strain showed the highest nitrogenase activity, with a C_2_H_2_ reduction rate of 83.06 nmol mL^−1^ h^−1^. Overall, the nitrogenase activities of these three strains of PSBdid not differ much (p > 0.05). Next, we tested the seven strains for their ACC deaminase activity. As shown in Table 5, three strains exhibited ACC deaminase activity ranging from 0.22 to 0.78 µmol mg^−1^ h^−1^. Among these, the ACC deaminase activity of strain HRP2 was the highest, at 0.78 µmol mg^−1^ h^−1^. Strains SSP2 and JRP22 exhibited similar ACC deaminase activities, at 0.22 µmol mg^−1^ h^−1^ and 0.50 µmol mg^−1^ h^−1^, respectively. Overall, the ACC deaminase activities of these three strains differed significantly (p < 0.05). Moreover, among the tested strains, five strains had the ability to produce IAA, at a concentration between 6.27 and 14.44 µg mL^−1^. Among these, strain JRP22 displayed the highest IAA production, followed by JLP2 (15.64 µg mL^−1^); the strain producing the lowest amount of IAA was SSP2 (6.27 µg mL^−1^). Most of the isolated PSBshowed the ability to produce IAA, which differed significantly among strains (p < 0.05). Finally, the siderophore production ability of the PSBwas tested by growth on Chrome azurol S (CAS) plates. The results showed that five strains could secrete iron carriers. Among these, JRP22 and HRP2 displayed ratios between the diameter of the siderophore-containing area and the colony diameter of 1.81, 0.91, which were significantly higher than those of the other strains (p < 0.05).

**Table 5:** Determination of the growth-promotion ability of phosphate-solubilizing bacteria.

Strain no	Soluble phosphorus content (mg L^−1^)	Nitrogenase activity (C_2_H_2_ nmol mL^−1^ h^−1^)	ACC deaminase (µmol mg^−1^ h^−1^)	IAA (µg mL^−1^)	Siderophore D/d
HRP2	123.73 ± 7.17a	83.06 ± 2.50a	0.78 ± 0.03a	7.64 ± 0.61c	0.91 ± 0.03b
JLP2	44.51 ± 2.2.52f	–	–	12.42 ± 0.67b	–
SSP2	116.51 ± 1.96b	76.09 ± 2.79b	0.22 ± 0.03c	6.27 ± 0.75c	0.88 ± 0.06b
JRP13	67.76 ± 2.26e	–	–	11.51 ± 0.82b	–
JRP22	79.36 ± 1.54c	73.19 ± 2.24b	0.50 ± 0.02b	14.44 ± 1.10a	1.81 ± 0.08a
JRP23	74.74 ± 1.67d	–	–	–	0.72 ± 0.03cf
JRP24	67.21 ± 1.67e	–	–	–	0.87 ± 0.05b

*ACC* 1-aminocyclopropane-1-carboxylate deaminase, *IAA* indole-3-acetic acid.

Significant differences among the seven bacterial strains for each growth promotion indicator were determined using one-way analysis of variance at p < 0.05. The data are shown as mean ± SD (n = 3).

Different lowercase letters represent significant differences among strains isolated from Chinese fir at p < 0.05.

### Effect of PSBs on Chinese fir growth

**Figure 2:**
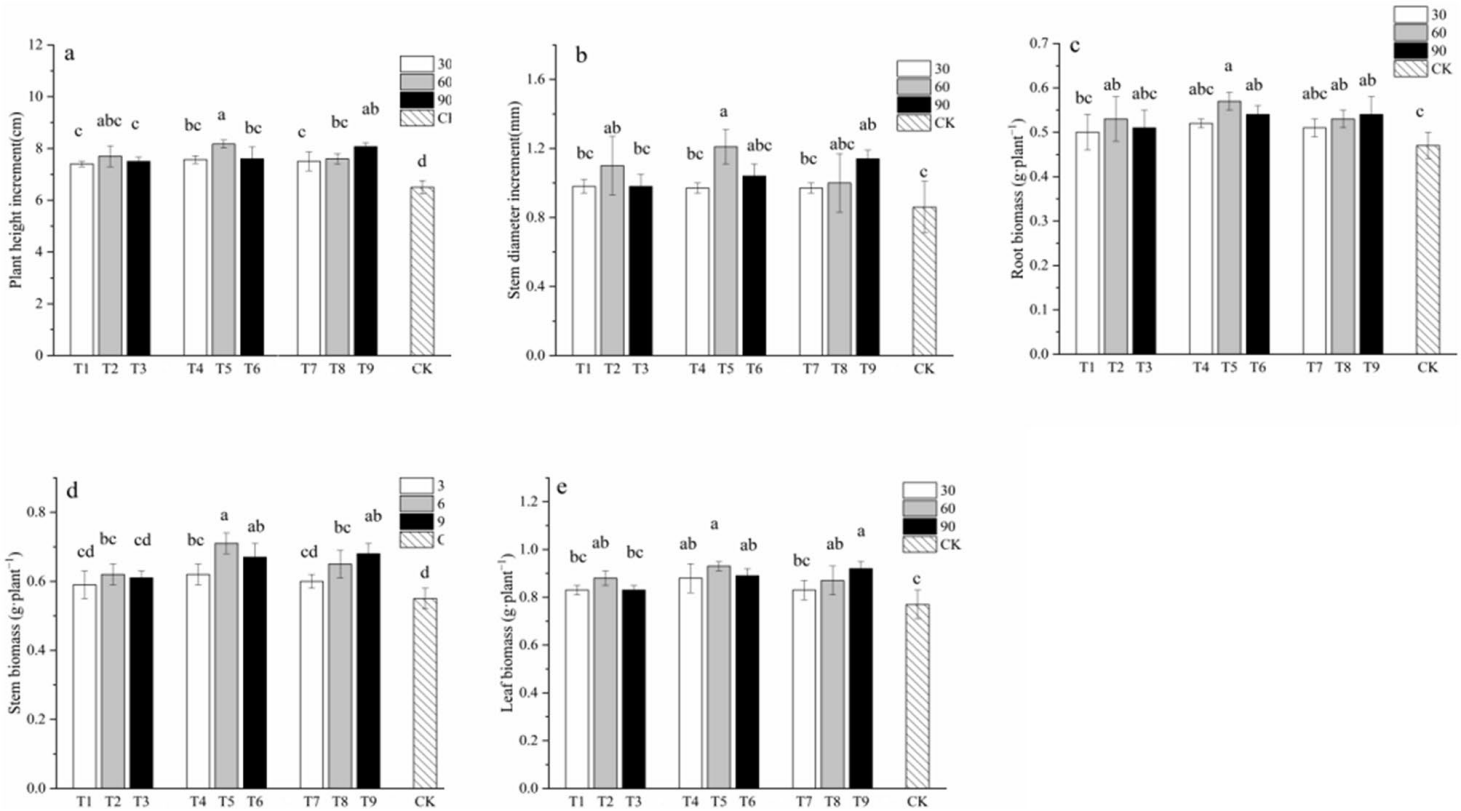
Growth response of Chinese fir seedlings inoculated with PSBs. (**a**) Plant height increment. (**b**) Stem diameter increment. (**c**) Root biomass. (**d**) Stem biomass. (**e**) Leaf biomass. Different lowercase letters represent significant differences at *p* < 0.05.

The three most promising phosphate-solubilizing bacterial strains, SP13, RP2, and RP22, were further examined for their growth-promoting activity for Chinese fir seedlings. Chinese fir seedlings were inoculated with increasingly diluted cultures (OD_600_ = 1.0, diluted by a factor of 30, 60, or 90) of strain SSP2 (seedlings T1, T2, and T3), strain JRP22 (seedlings T4, T5, and T6), and strain HRP2 (seedlings T7, T8, and T9). Compared to the CK, the inoculation with *Enterobacter hormaechei* SSP2, *Pseudomonas grimontii* JRP22, and *Pantoea roadsii* HRP2 significantly promoting Chinese fir seedlings growth (Fig. 2). Chinese fir seedlings inoculated with different strains exhibited significantly different increases in plant height (p < 0.05), which was, in all cases, significantly higher than that of control seedlings (p < 0.05). Among inoculated seedlings, T5 seedlings exhibited the highest increase in plant height (8.2 cm), which was about 1.26 times that of the control. T9 and T2 seedlings followed, with 1.24-fold and 1.18-fold increases plant height, respectively, as compared to that of control seedlings. Moreover, different treatments led to different growth-promoting effects on the ground diameter of Chinese fir seedlings (p < 0.05). Indeed, T5 seedlings showed the highest increase in ground diameter (1.21 mm), with an increase of 40.69% compared to that of the control, followed by T9 seedlings with an increase of 32.56% compared to that of the control. However, there was no significant difference in ground diameter growth of seedlings treated with the other dilutions of inocula compared with that of the control. Furthermore, compared to that of the control, the root dry weights of T5 and T9 seedlings both increased significantly (p < 0.05). In particular, the root dry weight of T5 seedlings was the highest, with an increase of 21.28% with respect to that of the control. In addition, the stem weights of T5, T6, and T9 seedlings were significantly higher than that of the control (p < 0.05), with increases of 29.09%, 21.82%, and 23.64%, respectively. Additionally, T5 and T9 seedlings displayed a significant increase in the dry weight of leaves (p < 0.05), which increased by 20.78% and 19.48%, respectively, compared with that of the control. In general, the quality of seedlings treated with strains JRP22 and HRP2 was higher than that of seedlings treated with strain SSP2. In addition, for inoculation with a single strain, dilution by a factor of 60 or 90 led to more significant increases in biomass (p < 0.05).

### Effect of PSBs on the nutrient content of Chinese fir

**Figure 3:**
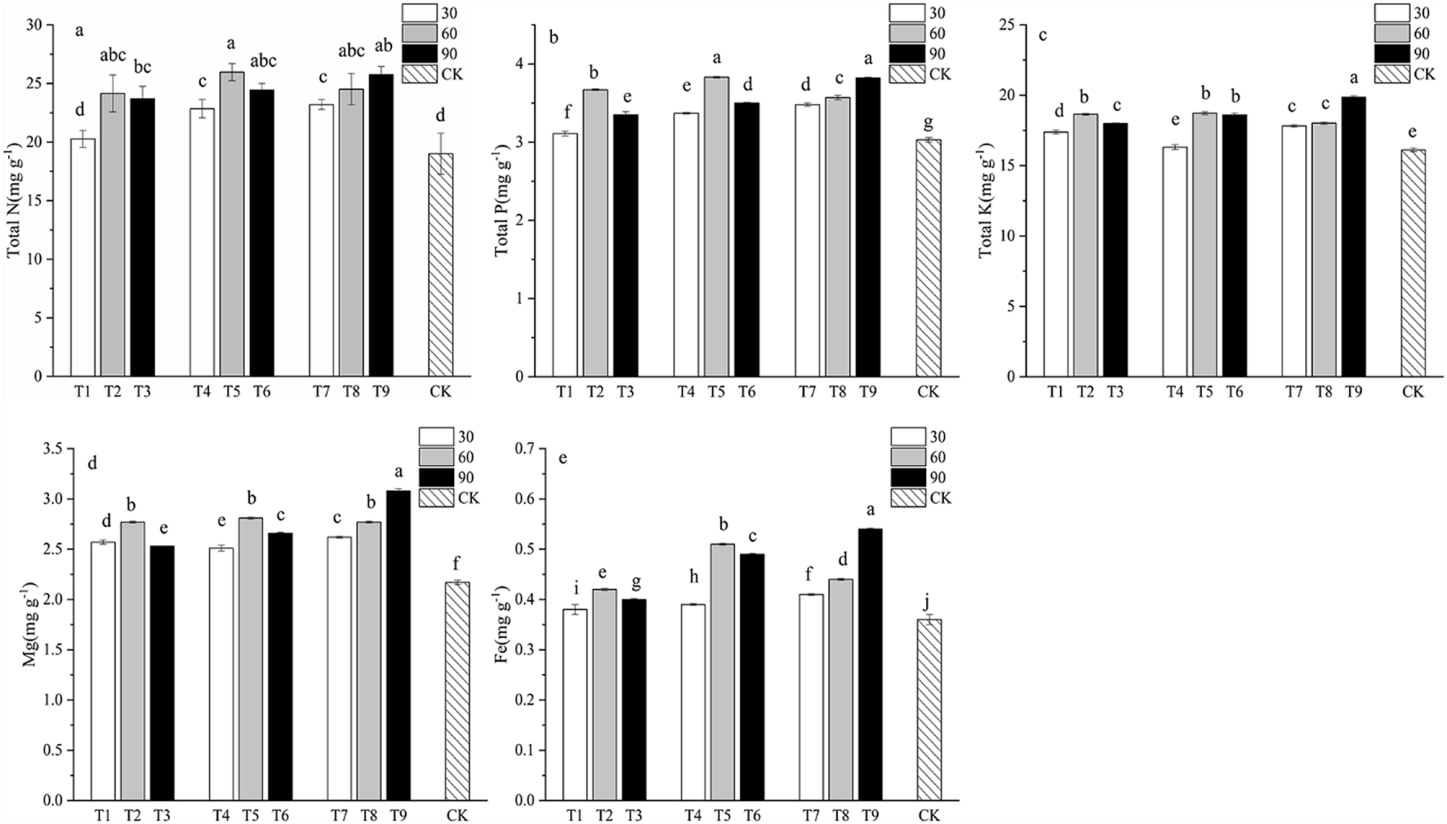
Leaf nutrient content of Chinese fir at 90 days after inoculation of PSBs. (**a**) Leaf total N content. (**b**) Leaf total P content. (**c**) Leaf total K content. (**d**) Leaf Mg content. (**e**) Leaf Fe content. Different lowercase letters represent significant differences at *p* < 0.05.

PSBs had a significant impact on the concentration of TN, TP, TK, Mg and Fe (Fig. 3). The T9 treatment significantly increased leaf TK, Mg and Fe content compared to the others treatments. The TN, TP, TK, Mg and Fe contents were significantly higher for T5 and T9 than for CK. The strain, dilution ratio and inoculation method had significant impact on the TN, TP concentration (Fig. 3a–c). All treatments, apart from inoculation with *Enterobacter hormaechei* SSP2 (T1) resulted in significantly increased TN content of Chinese fir compared to the control (CK). Compared to the treatment with dilution ratio of 1:30, the treatment with dilution ratio of 1:60 and 1:90 resulted in higher content of TN, TP, TK, Mg and Fe (Fig. 3a–c). The concentration of Mg and Fe significantly increased compared to CK (Fig. 3d–e).

### Nutrient content of soil

**Figure 4:**
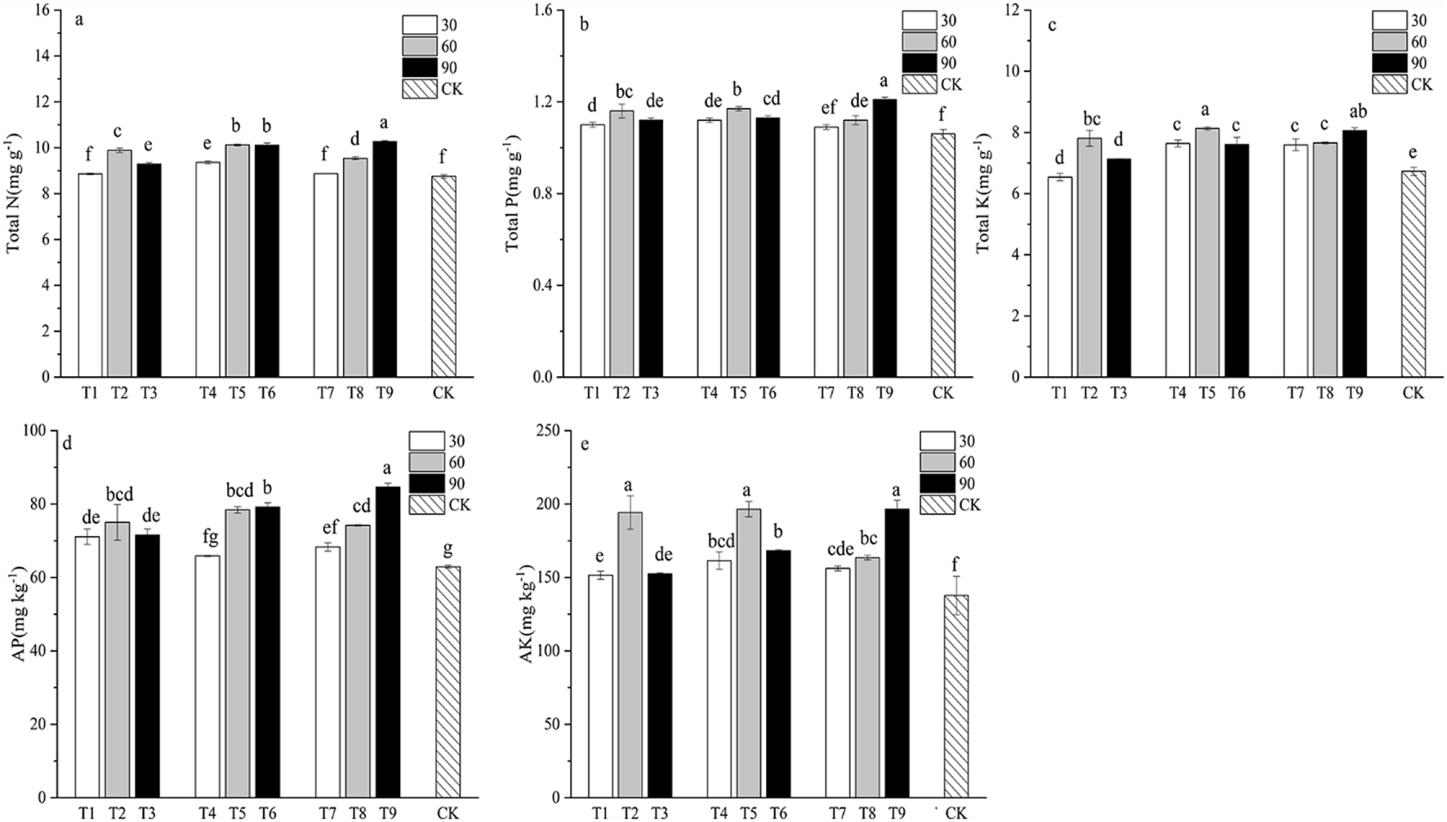
Soil nutrient content of Chinese fir at 90 days after inoculation of PSBs. (**a**) Soil total N content. (**b**) Soil total P content. (**c**) Soil total K content. (**d**) Soil AP content. (**e**) Soil AK content. Different lowercase letters represent significant differences at *p* < 0.05.

The PSBs significantly increased the nutrient content of soil compared with the control (Fig. 4). Similar to nutrient content of Chinese fir, inoculation with PSBs at the dilution ratio of 1:60 and 1:90 showed higher NPK content. Inoculation with HRP2 at the dilution ratio of 1:90 (T9) showed higher TN, TP, TK, AP and AK in soils than SSP2 and JRP22.

### Soil enzyme activities

**Figure 5:**
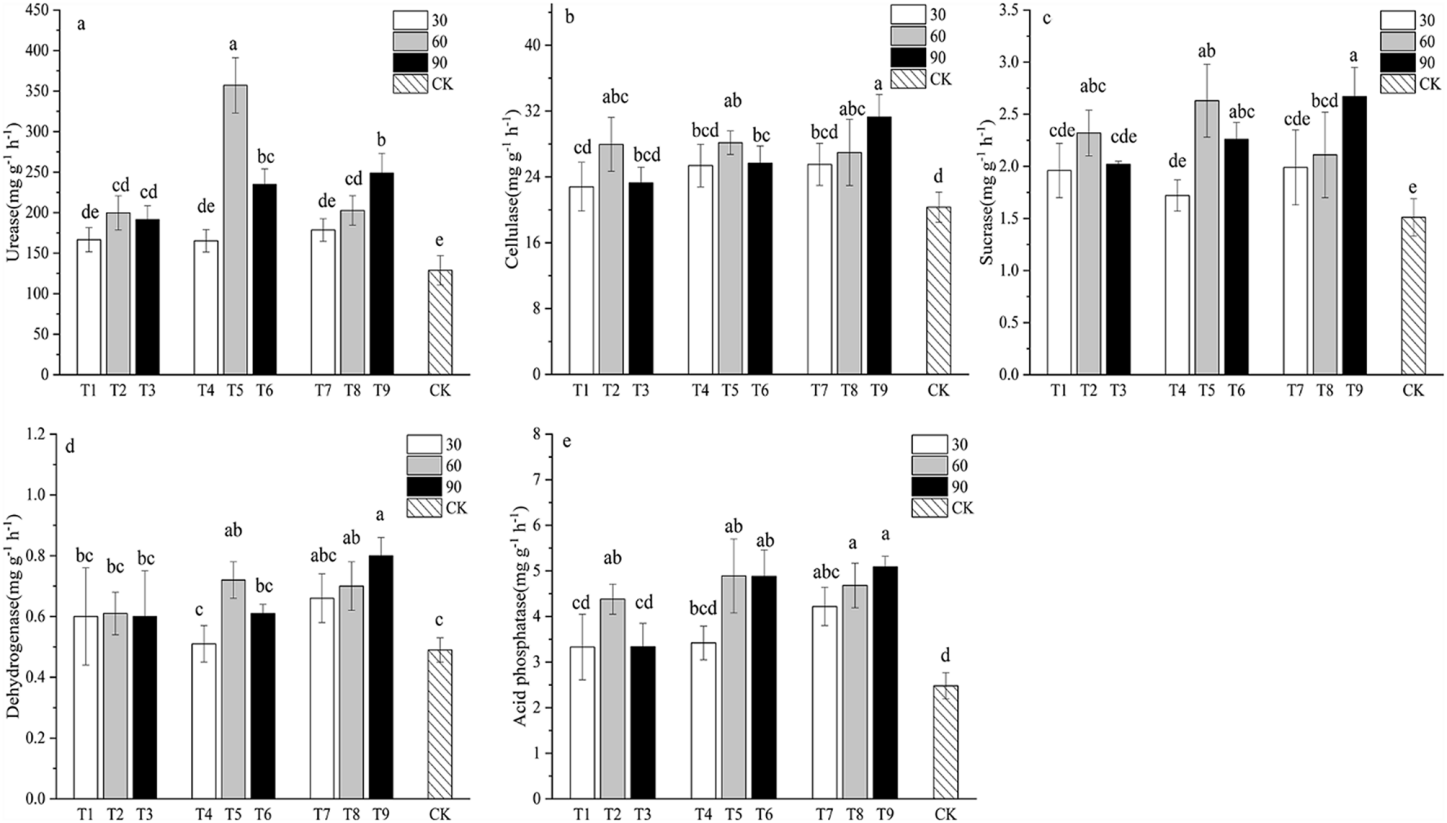
Soil enzyme activity at 90 days after inoculation of PSBs. (**a**) Soil total urease activity. (**b**) Soil cellulase activity. (**c**) Soil sucrase activity. (**d**) Soil dehydrogenase activity. (**e**) Soil acid phosphatase activity. Different lowercase letters represent significant differences at *p* < 0.05*.*

In this study, we found that soil urease, cellulase, sucrase, dehydrogenase and acid phosphatase activities all were influenced significantly by strain and dilution ratio (Fig. 5). Overall, inoculation of PSBs significantly increased the five enzyme activities. Strain and dilution ratio significantly influenced soil urease and acid phosphatase activity, whereas inoculation method had not-significant effects on the six enzyme activities. The urease activity was highest in T5 inoculated with JRP22 at the dilution ratio of 1:60 (Fig. 5a). The other five enzyme activities were highest in T9.

## Discussion

Among the PSBs isolated in this study, the number of *Pseudomonas* strains was the highest. Currently known phosphorolytic bacteria mainly include species of the genera *Burkholderia*, *Bacillus*, *Pseudomonas*, *Erwinia*, and *Klebsiella*, among which *Pseudomonas*, *Burkholderia*, and *Bacillus* have been extensively studied^32, 33^. The phosphate solubilization abilities of the strains isolated and screened in this study ranged from 44.29 to 195.61 mg L^−1^. These were significantly higher than the phosphate solubilization abilities of bacteria isolated from the roots of Moroccan wheat and grown in liquid medium, which ranged from 59.1 to 90.2 µg mL^−1^^34^. On the contrary, Qiao et al.^35^ found that phosphorus-solubilizing bacteria from maize rhizosphere soil could dissolve phosphorus up to 487.67 mg L^−1^ and proposed that the ability of each strain was related to its physiological and metabolic characteristics. Studies have shown that fungi are more efficient than bacteria at solubilizing phosphate. Indeed, the phosphate solubilization ability of most bacteria ranges from 26.9 to 43.3 mg L^−1^, while phosphate solubilization ability of fungi is generally 3.2 to 5.4 times higher than that of bacteria (59.6 to 145.4 mg L^−1^)^36, 37^. Interestingly, the HRP2, SSP2, and JRP22 bacterial strains isolated in this study displayed a phosphate solubilization ability in the range of those of fungi and 2.9–7.2 times higher than the average ability of bacteria. This result indicates that these three strains are very good at solubilizing phosphate, with the HRP2 strain reaching an activity of up to 195.61 mg L^−1^. Moreover, in this study we found that the seven isolated strains possessed ACC deaminase activity, ranging from 0.2 to 0.74 µmol mg^−1^ h^−1^, consistent with the results reported by Han et al.^38^. Among them, strain HRP2 (*Burkholderia ubonensis*) exhibited the highest ACC deaminase activity (0.74 µmol mg^−1^ h^−1^), which was higher than that of the *B. ubonensis* strain CBPB (0.21 µmol mg^−1^ h^−1^) isolated from the rhizosphere of rice previously reported by Poonguzhali et al.^39^. Such a difference may be related to the soil type and the type of host plant from which strains were isolated. In fact, different habitats can greatly influence the physiological and metabolic processes of a bacterial strain^40^. IAA is an important plant hormone that participates in many important physiological processes in plants, including cell growth and division, and tissue differentiation, thereby promoting the growth of plant roots and shoots^41, 42^. Among the strains tested in this study, strain HRP2 displayed the strongest IAA production capacity (18.00 µg mL^−1^), although this was quite lower than that of *B. ubonensis* CBPB-HIM (28.02 µg mL^−1^) previously reported by Poonguzhali et al.^39^ but similar to those of the isolates characterized by Abderrazak et al.^32^, which exhibited IAA production capacities of 2.9–21.2 µg mL^−1^. Siderophores are small organic compounds produced by microorganisms under iron deficiency, which gather insoluble iron compounds from the environment and convert them into a form that can be absorbed and utilized by plants^43^. Therefore, siderophores promote plant growth by enhancing iron absorption by plants^44^. Among the strains tested in this study, strain JRP22 (*P. grimontii*) showed the highest production of siderophores, with an iron carrier circle diameter to colony diameter ratio of 1.80. On the contrary, Chen et al.^45^ found seven *Pseudomonas* isolates producing iron carriers with an iron carrier circle diameter to colony diameter ratio of 1.50–12.00. These findings indicate that *Pseudomonas* strains possess a highly variable siderophore production ability. Some studies have pointed out that the ability of bacterial strains to produce siderophores is mainly related to their genetic characteristics, different strains secrete different types of siderophores depending on the degree of iron chelation in the soil^43^.

Studying the influence of PSB on the morphological and physiological characteristics of Chinese fir seedlings and thereby unraveling the growth-promoting properties of these bacteria can prove useful for the application of PSB to forest trees. Plant height, ground diameter, biomass, and other morphological growth indicators are a direct manifestation of the efficiency of seedling growth. The results of this study showed that phosphorus-solubilizing bacteria promote the growth of Chinese fir seedlings in terms of plant height, ground diameter, and root, stem, and leaf biomass to varying degrees. This might be due to the PSB strains dissolving the insoluble phosphate in the soil and enhancing the available P content by producing organic acid and extracellular phosphatases^46, 47^. Another possibility might be related to the metabolism of PSB, producing a variety of plant hormones, acids, and vitamins^48^.This is consistent with the findings of Cui et al.^49^ and Xue et al.^50^, who demonstrated that phosphorus-solubilizing bacteria can promote plant growth and increase rhizome and leaf biomass. The observed increases in plant height, ground diameter, and biomass are likely due to the ability of PSB to dissolve phosphorus and nitrogen, which promote efficient absorption and use of nutrients to synthesize organic matter and increase plant biomass. Similarly, Pereira et al.^51^ found that the inoculation of PSB significantly promoted plant height, ground diameter, and biomass, which can be due to the organic acids production (such as gluconic, formic, and citric acids) by these strains. In this study, the plant height growth and biomass of seedlings treated with bacteria were significantly higher than those of control seedlings. This may be due to the fact that the seedlings used in this study were sown in the same year, and most of the nutrients in the seedlings thus flowed to the roots and leaves^52^. The results of this study showed that the growth-promoting effects of the strains JRP22 and HRP2 were stronger than that of strain SSP2; this phenomenon may depend on differences in growth-promoting characteristics and environmental adaptability among these strains. When plants were treated with the same strain, higher dilutions (by a factor of 60 or 90) triggered a more obvious growth promotion effect. Indeed, the plant height, ground diameter, and stem and leaf dry weight of seedlings treated with low bacterial concentrations were significantly higher than those of plants treated with high bacterial concentrations. This finding is consistent with the results of Xu et al.^53^ and of Luan et al.^54^ in red bean. Altogether, these results indicate that phosphorus-solubilizing bacteria can promote plant growth within a certain range of concentrations. It turns out that the mechanism of promoting growth is a complex and comprehensive effect.

Soil nitrogen, phosphorus and potassium content is considered to be an important indicator of soil fertility, which reflects the storage and supply of nutrients in the soil^55^. PSBs increase the content of nitrogen and phosphorus in soil by dissolving insoluble phosphate in the soil and fixing nitrogen^56, 57^. PSB not only solubilize and mineralize P from insoluble compounds but also release other nutrients^58, 59^. This study found that the effect of PSBs on soil nutrient content was significantly different. Compared to the control, the content of TN, TP, TK, AP and AK increased significantly, demonstrating that PSB elevated the amounts of available N, P, and K in the soil and subsequently provided better nutrition for plant growth^60, 61^, which is consistent with the results of Matsumura et al.^62^ and Wang et al.^63^ found that inoculation with PSBs significantly increased the accumulation of available phosphorus and nitrogen in the soil, and effectively promoted the growth of Willow seedlings.

Soil enzymes are catalysts for biochemical reactions in soil and it affects microbial activity in the soil and the characteristics of biochemical processes^64, 65^. The results of this study indicated that PSBs significantly increase the enzyme activity of soil. This may be because the organic acids secreted by the strains during the growth and metabolism process lead to the decrease of soil pH, which is more conducive to the increase of soil enzyme activity^66^. Khati et al.^67^ studied the response mechanism of corn to phosphate-dissolving bacteria and found that soil enzyme activity increased by 2 to 3 times. Soil cellulase and soil dehydrogenase are closely related to microbial biomass^56, 68, 69^. This study showed that the cellulase and dehydrogenase activity of T9 was the highest, indicating that the treatment had a large number of substrate microorganisms. Shahzad^70^ reported that PSBs significantly increased the activity of soil dehydrogenase, beta-glucosidase, and urease.

## Conclusion

Seven strains of phosphorus-solubilizing bacteria cloud be isolated from the roots, stems, and leaves of Chinese fir. The isolated strains were characterized and identified by analysis of colony characteristics, physiological and biochemical testing, and phylogenetic analysis based on 16S rRNA gene sequencing. Found that these seven phosphate solubilizing bacteria belong to the genus *Pseudomonas*, *Pantoea*, *Enterobacter*, *Paraburkholderia*, *Novosphingobium*, *Ochrobactrum.* Next, their growth-promoting characteristics, such as their ability to fix nitrogen, produce IAA, and secrete siderophores, were studied. Interestingly, the phosphate solubilization ability of the bacterial strain HRP2 reached the level of the phosphate solubilization ability of fungi. The strain showing the highest nitrogenase activity was SSP2, while the best iron carrier producer was JRP22. In the growth promotion test, compared with the control, PSBs significantly improved the growth of Chinese fir seedlings, including plant height, stem diameter, biomass, and nutrient content of Chinese fir. At the same time, soil nutrient content and enzyme activity were significantly increased, such as total N, total P, total K, AP, AK, soil urease, cellulase, sucrase, dehydrogenase, nitrate reductase and acid phosphatase. Increased enzyme activity was significantly associated with increased nutrient content. In the future, these isolated and identified strains can be tested for the presence of virulence factors to understand the influence of the tested strains on the composition and richness of endophytic microbial communities to fully evaluate the application potential of these strains as microbial fertilizers in agroforestry.
